# The Articular Chromatin Landscape in Osteoarthritis

**DOI:** 10.3390/cells14201600

**Published:** 2025-10-15

**Authors:** George D. Kalliolias, Efthimia K. Basdra, Athanasios G. Papavassiliou

**Affiliations:** Department of Biological Chemistry, Medical School, National and Kapodistrian University of Athens, 11527 Athens, Greece; kallioliasg@med.uoa.gr (G.D.K.); ebasdra@med.uoa.gr (E.K.B.)

**Keywords:** osteoarthritis, chromatin, epigenetics, enhanceropathies

## Abstract

**Highlights:**

**What are the main findings?**

- Emerging data from multidimensional and high-resolution phenotyping of disease-relevant tissues indicates that osteoarthritis (OA) is a polygenic disease, where genetic factors disrupt the chromatin landscape in disease-relevant cells leading to aberrant expression of effector genes that drive OA pathogenesis.
- According to the concept of a developmental origin for OA, the functional cooperation between chromatin dynamics and transcription factors (TFs) regulates the unfolding of a development-specific gene expression program, defines the outcome of skeletogenesis, and ultimately determines the articular biomechanics and the risk of OA.

**What is the implication of the main findings?**

- Detailed mapping and functional characterization of the OA-associated chromatin conformation and epigenetic disruptions may accelerate the discovery of disease-modifying drugs for OA.
- Novel technologies pave the way for precise epigenetic editing at the desired genomic regions and may allow a targeted transcriptional regulation of OA-relevant genes in disease-relevant cells.

**Abstract:**

Recent technological breakthroughs have enabled multidimensional phenotyping, with unprecedented single-cell resolution and genome-wide coverage, across multiple osteoarthritis (OA)-relevant tissues, such as articular cartilage, synovium, infrapatellar fat pad, and subchondral bone. The majority of the single nucleotide variations (SNVs) that have been associated with OA are located in non-protein coding regions and confer risk for disease by altering the expression level, instead of the amino acid sequence of the gene product. These data have shaped the concept of OA as a polygenic disease, where genetic factors disrupt the chromatin landscape in disease-relevant cells, leading to aberrant expression of effector genes. Pharmacologic manipulation of the OA-driving epigenetic landscape has recently emerged as an attractive path for the development of disease-modifying drugs. Novel clustered regulatory interspaced short palindromic repeats (CRISPR)-based technologies provide opportunities for precise epigenetic editing at the desired genomic regions and may allow a targeted transcriptional regulation of disease-relevant genes in disease-relevant cells. The aim of the present narrative review is to summarize the emerging data on the role of epigenetic factors and chromatin structure as calibrators of the risk for developing OA and to discuss the opportunities and challenges arising from the use of chromatin landscape to guide drug discovery.

## 1. Introduction

Osteoarthritis (OA) is the most common type of arthritis, imposing a substantial socioeconomic burden due to its prevalence, impact on quality of life, and associated costs [1]. Based on the current trends in population growth, aging, and obesity, it is estimated that nearly one billion people worldwide will be living with OA by 2050 [2]. Despite many years of investigation, there are no disease-modifying drugs approved yet for OA [3,4]. Symptom-relieving approaches, including pharmacologic management of pain and life-style modifications, represent the current therapeutic paradigm. A substantial proportion of patients with end-stage OA, finally require surgical replacement of the affected joint [1]. In this context, there is a pressing need for a better understanding of the molecular mechanisms involved in the pathophysiology of OA, that will hopefully pave the way for the development of novel and rational treatments aiming to halt disease progression [5].

To fulfill the requirement for a deeper understanding of OA pathogenesis, a series of new approaches have been implemented [6]. At first, the field has shifted from the traditional “cartilage-centric” view to a more holistic approach, where OA is considered a whole-joint disease [7]. Although cartilage degradation still remains the hallmark of OA, researchers have recently focused on additional disease features, such as synovial and infrapatellar fat pad inflammation, subchondral bone thickening, osteophyte formation, meniscal degeneration, and ligament degeneration [8,9,10]. In this context, the molecular phenotyping of disease-relevant tissues now goes beyond chondrocytes and extracellular matrix (ECM) of the articular cartilage, including synovial tissue, infrapatellar fat pad, and subchondral bone [11]. It is of note that, in OA, certain of the above-mentioned tissues operate as integrated anatomo-functional units, specifically, the infrapatellar fat pad with synovium, and bone with cartilage [12,13]. In addition, it has been appreciated that, for the meaningful interpretation of molecular phenotyping data, it is necessary to account for multiple factors, including but not limited to disease heterogeneity (e.g., weight-bearing joint OA vs. non-weight-bearing joint OA), disease stage (e.g., early OA vs. end-stage OA), and sex (female OA vs. male OA) [14].

The research of OA pathogenesis has been bolstered recently by the shift in cellular and molecular profiling approaches from “bulk” sequencing and “gene-centric” methods to next-generation sequencing (NGS), single-cell and single-nuclei technologies, multi-omic analyses, and “team-science” approaches [15,16,17,18]. These breakthroughs have allowed a multidimensional phenotyping of OA-relevant cells, with unprecedented single-cell resolution and genome-wide coverage [19]. In addition, recent large-scale meta-analyses of genome-wide association studies (GWASs) have expanded the list of single nucleotide variations (SNVs) that have been associated with OA [20,21,22]. These massive datasets, including data from OA-relevant cell types on gene and protein expression, chromatin accessibility and three-dimensional (3D) structure, DNA methylation, and histone post-translation modifications (PTMs), combined with the data on the OA-associated SNVs, offer the opportunity for variant-to-function analyses that can lead to the discovery of OA-promoting cellular and molecular pathways and the development of disease-modifying treatments [19,23,24]. Herein, we summarize the emerging data on the role of epigenetic factors (focusing on DNA methylation and chromatin structure) as calibrators of the risk for developing OA and discuss the opportunities and challenges arising from the use of chromatin landscape to guide drug discovery for OA.

## 2. GWASs Implicate Epigenetic Regulation and Chromatin Structure in OA Pathogenesis

Data on the genetic architecture of OA reveal a potential role of epigenetic factors in disease pathogenesis. In the last few years, genetic consortia focused on OA have conducted large-scale meta-analyses by pooling GWAS data from multiple sources that have firmly placed OA in the polygenic category of common diseases [20,21,22,23]. There is a continuously rising number of SNVs found to be statistically and functionally associated with OA, each one exerting a modest effect. According to the most recent meta-analysis, the OA-associated SNVs discovered so far are linked to the function of up to 700 genes [22]. Fine-mapping in the genome indicates that the majority of the OA-associated SNVs are located in non-protein coding regions, such as the promoters, large intergenic spaces, and the intronic regions of genes [25]. These SNVs, that are mapped in non-protein coding regions, may influence the function of the associated genes by altering the expression level, instead of the amino acid sequence of the gene product [26].

The impact of OA-associated SNVs on the levels of gene expression in disease-relevant cells (mainly chondrocytes) has been confirmed by several studies using two different approaches known as allelic expression imbalance (AEI) and expression quantitative trait loci (eQTL) analysis. AEI is performed in heterozygous individuals who carry the SNV in one allele and compares, in the same individual, the mRNA levels derived from each allele [27]. AEI aims to capture an imbalance in the relative contribution between the two alleles, and any deviation from a 1:1 ratio in mRNA levels indicates a differential contribution of the SNV-bearing allele in the total gene expression. AEI analysis has confirmed the impact of OA risk-conferring SNVs in lowering the articular expression of *aldehyde dehydrogenase 1 family member A2* (*ALDH1A2*), *matrix Gla protein* (*MGP*), and *plectin* (*PLEC*) genes [28,29,30]. *ALDH1A2* encodes a key enzyme that catalyzes the synthesis of all-trans retinoic acid (ATRA) that regulates cartilage homeostasis and response to injury by modulating the expression of chondrogenic and inflammatory genes [31]. MGP is a vitamin K-dependent protein that prevents ectopic calcification by inhibiting calcium phosphate deposition in the ECM of cartilage [29]. Finally, *PLEC* encodes a cytoskeletal protein that regulates response of chondrocytes to mechanical stress by crosslinking components of the cytoskeleton and by operating as a scaffolding molecule involved in intracellular signaling [30].

On the other hand, eQTL analysis aims to capture any correlation between an SNV and the expression level of a gene by comparing mRNA levels of the given gene among a cohort of individuals grouped by their genotype at the investigated SNV [19]. Application of the eQTL approach in chondrocytes derived from a cohort of over 100 patients undergoing joint replacement for OA, resulted in the discovery and prioritization of five effector genes (eGenes) including *ALDH1A2*, *Niemann–Pick disease type C1* (*NPC1*), *family with sequence similarity 53 member A* (*FAM53A*), *mothers against decapentaplegic homolog 3* (*SMAD3*), and *solute carrier family 44 member 2* (*SLC44A2*) [32,33]. Notably, for all five genes, the OA-associated SNVs reside in non-coding regions [32].

**Figure 1:**
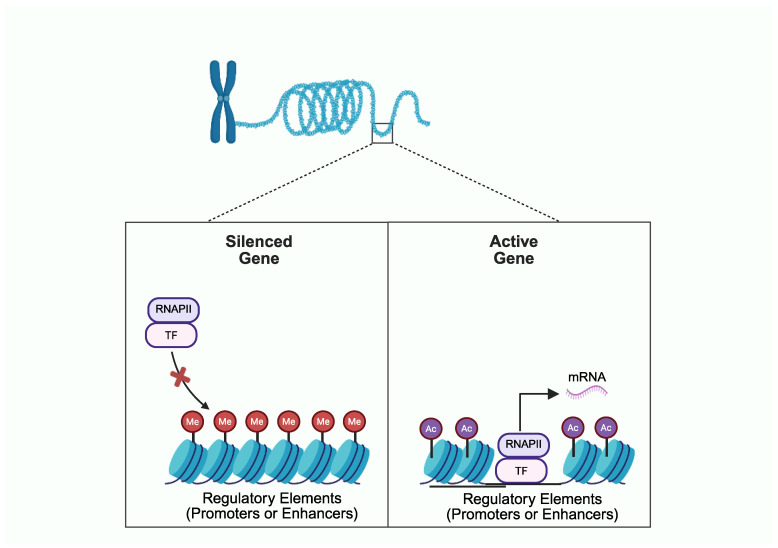
Chromatin architecture and epigenetic modifications regulate the rate of gene expression. Left panel: regulatory elements (REs) of silenced genes display CpG hypermethylation (Me), low levels of histone acetylation, and tightly packed chromatin, preventing the binding of transcription factors (TF) and RNA polymerase II (RNAPII). Right panel: REs of active genes display highly accessible chromatin to TFs and RNAPII due to high levels of histone acetylation (Ac) and histone depletion. Created in BioRender (https://BioRender.com/4x5v8p6; accessed on 28 September 2025).

**Figure 2:**
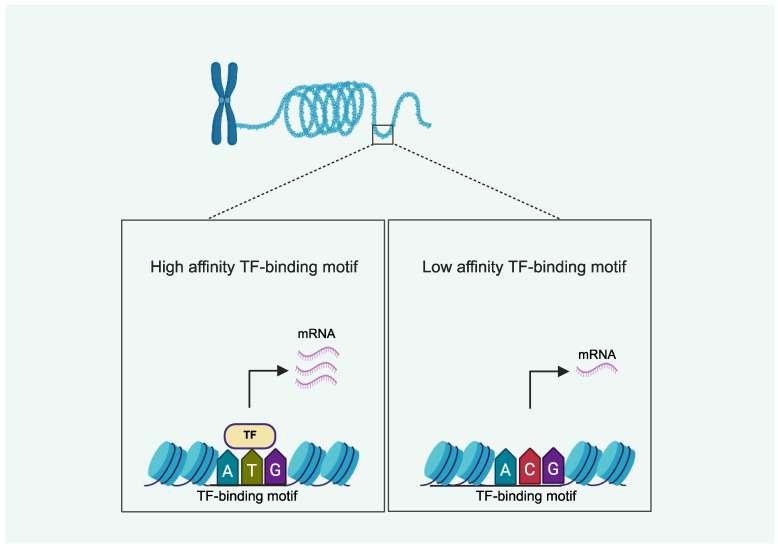
Single nucleotide variations (SNVs) associated with osteoarthritis (OA) may alter the rate of gene expression via direct effects on binding affinity of transcription factor (TF)-binding motifs. Left panel displays a high affinity binding-motif that drives robust gene expression. The right panel illustrates an SNV (T to C), which lowers the affinity for TF binding and hampers gene expression. Created in BioRender (https://BioRender.com/phnlj1k; accessed on 28 September 2025).

The rate of gene expression is tightly controlled by epigenetic mechanisms, which impact the chromatin architecture and regulate the recruitment and function of the transcriptional machinery, without changing the DNA sequence [34]. Chromatin accessibility, DNA methylation at cytosine–guanine dinucleotides (CpGs), and histone PTMs at genomic regions known as regulatory elements (REs) are three key epigenetic regulators of gene expression (Figure 1) [24,25]. Promoters are REs proximal to a gene’s transcriptional start site (TSS), and enhancers are REs that typically reside within non-coding intergenic or intronic regions. Promoters and enhancers consist of numerous transcription factor (TF)-binding sites that regulate gene expression [25]. OA-associated SNVs may influence the function of REs and alter gene expression via direct or indirect effects on TF binding. SNVs mapped within TF-binding motifs may directly alter the binding affinity of TFs on REs (Figure 2). For example, one OA-associated SNV (i.e., the T allele at rs6060369) was found to be located at a TF-binding site within an enhancer region, disrupting the binding of pituitary homeobox 1 (PITX1) and downregulating the expression of *growth differentiation factor five* (*GDF5*) [35]. Alternatively, since the status of CpG methylation in REs leads to differential TF binding, and histone acetylation increases accessibility for TFs, SNVs may alter TF binding indirectly by modulating DNA methylation or histone acetylation [24,25].

## 3. Methodological Advances Enabling Study and Interpretation of the OA Chromatin Landscape

The concept of OA as a polygenic disease, where genetic factors disrupt the chromatin landscape in disease-relevant cells leading to aberrant expression of eGenes, has been shaped recently as a result of elegant studies applying novel technologies that capture multiple dimensions of chromatin structure [22,33,35]. The “toolkit” for capturing nucleosome-depleted regions and evaluating chromatin accessibility includes the assay for transposase-accessible chromatin with high-throughput sequencing (ATAC-seq), formaldehyde-assisted isolation of regulatory elements with sequencing (FAIRE-seq), and DNase I hypersensitivity sites with sequencing (DNase-seq) [36,37]. High-throughput detection of chromatin interactions is now feasible with the application of chromatin interaction analysis by paired-end tag sequencing (ChIA-PET) and chromatin conformation capture technologies such as high-throughput chromosome conformation capture (Hi-C), 3C, and Capture-C [38,39]. Bisulfite conversion-based technologies, such as whole genome bisulfite sequencing (WGBS) and reduced representation bisulfite sequencing (RRBS), are the gold standard for measuring DNA methylation [40]. Additional technologies to study DNA methylation are also available, offering varying levels of resolution, cost, and genome coverage. Finally, genome-wide mapping of histone PTMs (e.g., acetylation and methylation) is enabled by chromatin immunoprecipitation (ChIP)-based methods, cleavage under targets and release using nuclease (CUT&RUN), and cleavage under targets and tagmentation (CUT&Tag) [41,42,43,44].

A combination of the above-mentioned technologies offers the opportunity for the generation of large-scale data that provides a multidimensional profiling of gene expression, protein abundance, chromatin states, and epigenetic modifications in disease-relevant tissues, such as articular cartilage, synovium, and subchondral bone. Interpretation of large-scale data and translation into clinically actionable information is challenging [45]. To address this challenge, an integrated approach has been implemented, where different sets of data capturing multiple dimensions of the chromatin landscape are analyzed together with genetic data and genome-wide maps of multiple molecular traits (i.e., DNA methylation QTLs (mQTLs), protein QTLs (pQTLs), and eQTLs) [22,32,33,35,46,47]. A recently published excellent application of this approach used integrated analysis of 24 orthogonal lines of information, including OA-associated variant information and datasets on chromatin conformations, DNA methylation, histone PTMs, gene expression, and protein abundance across multiple OA-relevant cell types [22]. This sophisticated type of integrated analysis has mapped causal variants in REs and within enhancers that loop to promoters. In addition, the study team has used the above-mentioned 24 orthogonal lines of information to score each candidate gene, and this scoring approach has led to the discovery of 700 putative eGenes distributed across multiple biological pathways, including retinoic acid signaling, transforming growth factor beta (TGFb) signaling, bone morphogenetic protein (BMP) signaling, fibroblast growth factor (FGF) signaling, ECM assembly and organization, circadian rhythm, and glial cell-related pathways. The highest-scoring eGene, with eleven lines of evidence supporting its involvement in OA pathogenesis, was *ALDH1A2*.

## 4. Unraveling the DNA Methylome Landscape in OA

DNA methylation is the most extensively studied epigenetic modification in OA. It involves the covalent addition of a methyl group (-CH_3_) in cytosines (C) at genomic regions where C is followed by a guanine (G), forming dinucleotides known as CpGs [48]. The reverse process involves the chemical conversion of 5-methylcytosine (5-mC) to 5-hydroxymethylcytosine (5-hmC). The enzymatic machinery that regulates methylation status of CpGs primarily comprises three DNA methyltransferases (DNMT1, 3A, and 3B) and three demethylases from the family of ten-eleven translocation (TET) enzymes (TET1-3). In the human genome, there are approximately 28 million CpGs. The location, density (isolated CpGs vs. long CpG repeats known as CpG islands), and methylation status of CpGs play critical roles in gene expression regulation. Methylation of CpGs around the TSS or within enhancers of genes tend to repress gene expression, through the recruitment of repressive methyl-binding proteins that prevent the binding of transcriptional activators. DNA methylation within the gene body is less predictable and may have gene-specific functional consequences [48].

**Figure 3:**
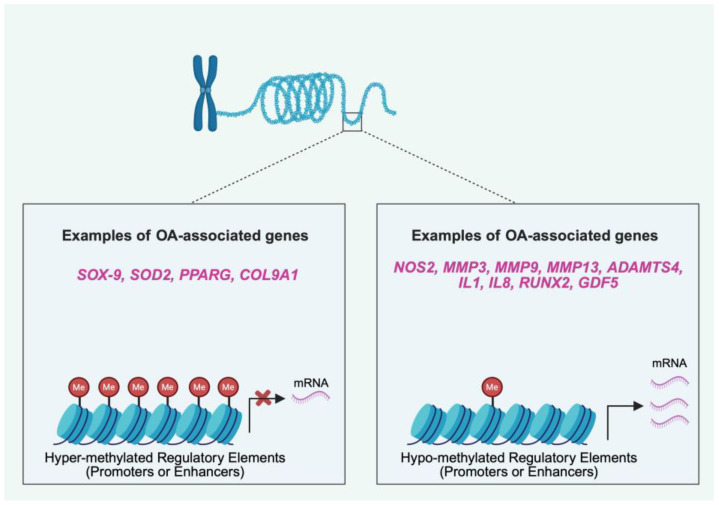
DNA methylation status as a calibrator of gene expression for specific effector genes (eGenes) during the pathogenesis of OA. Methylation of CpGs around the transcription start site (TSS) or within enhancers of genes tend to repress gene expression, through the recruitment of repressive methyl-binding proteins that prevent the binding of transcriptional activators. Examples of eGenes with hyper-methylated regulatory elements and decreased expression in OA include *SOX-9*, *superoxide dismutase 2 (SOD2)*, *peroxisome proliferator-activated receptor gamma* (*PPRG*), and *collagene type IX alpha 1 chain* (*COL9A1*). Down regulation of DNA methylation at regulatory elements is associated with increased gene expression. Examples of eGenes with hypo-methylated regulatory elements and increased expression in OA include *nitric oxide synthase 2* (*NOS2*), *matrix metallopeptidase 3* (*MMP3*), *matrix metallopeptidase 9* (*MMP9*), *matrix metallopeptidase 13* (*MMP13*), *a disintegrin and metalloproteinase with thrombospondin motifs 4* (*ADAMTS4*), *interleukin 1* (*IL1*), *interleukin 8 (IL8)*, *ruant-related transcription factor 2 (RUNX2)*, and *growth differentiation factor five* (*GDF5*). Created in BioRender (https://BioRender.com/l9gsfzz; accessed on 28 September 2025).

Initial genome-wide studies in humans have shown distinct DNA methylation signatures in tissues from affected joints of OA patients [49,50,51,52,53,54,55,56]. The potential functional consequences of the differentially methylated CpGs in OA have been explored by a series of gene-targeted approaches showing an impact on gene expression for specific susceptibility genes, such as *GDF5* and *SOX-9* [57,58]. These observations have triggered the generation of genome-wide maps of the differentially methylated CpGs and the analysis for potential functional correlations between DNA methylation status, gene expression, and OA-associated SNVs [35,46,47,59,60]. Up to 25% of the OA-associated SNVs have been reported to be correlated with altered levels of DNA methylation in chondrocytes [59]. For SNVs that are proximal to differentially methylated CpGs, one potential mechanistic explanation is that the SNVs regulate the recruitment of DNMTs [25]. Notably, for some genes (e.g., *GDF5* and *ALDH1A2*), it was shown that particular SNVs were correlated with two molecular layers, differential DNA methylation and gene expression [60]. This finding suggests that DNA methylation status is a critical calibrator of gene expression for specific eGenes during the pathogenesis of OA (Figure 3), and for some SNVs, operates as a potentially druggable intermediate between disease-predisposing genotype and disease development.

The construction of cell type-specific genome-wide methylation quantitative trait loci (mQTL) maps for primary cells from patients with OA enabled the systematic evaluation of DNA methylation in articular cartilage, synovium, subchondral bone, infrapatellar fat pad, and peripheral blood [35,46,47,59,60]. Recently emerged evidence from these datasets revealed cell type, disease stage, and joint location specificity for DNA methylation signatures [35,47,59]. Several OA-associated SNVs were correlated with CpG methylation changes only in chondrocytes or infrapatellar fat pad adipocytes but not in peripheral blood cells, suggesting that the disease-predisposing SNVs mediate their effects on DNA methylation exclusively in disease-relevant cells. This observation emphasizes the requirement for studies focused on disease-relevant cell types and tissues (e.g., articular cartilage, synovium, subchondral bone, and infrapatellar fat pad). Notably, although there were shared OA-associated signals in cartilage, synovium, and subchondral bone, cell-type-specificity in disease-associated DNA methylation signatures was also observed within the different disease-relevant tissues, with some signals specifically observed, for example, only in cartilage but not in synovium and subchondral bone. Overall, these findings indicate that studying the cell type-specific epigenetic landscape of disease-relevant cells may reveal the cell type-specific contributions in OA pathogenesis and pave the way for the discovery of targeted disease-modifying drugs. Equally important was the observation that between low- and high-grade OA there were 18 differential methylation QTLs (mQTLs) in the articular cartilage, indicating a disease stage-specific impact of genetic predisposition and DNA methylation status [47]. Finally, the identification of differential cartilage DNA methylation profiles between knee and hip OA indicates joint type-specificity in the disease-associated epigenetic landscape [51,54].

## 5. Chromatin Structure as a Calibrator for Gene Expression Defining the Risk for OA

**Figure 4:**
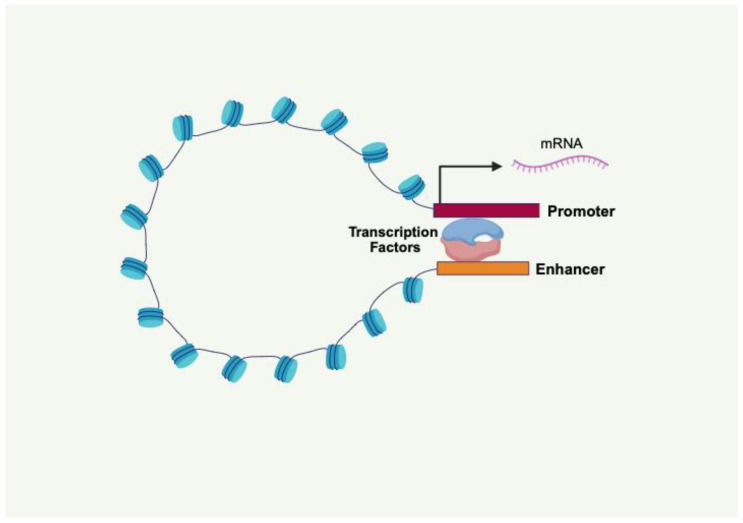
Enhancers amplify the recruitment of the transcriptional machinery and increase gene expression by physical interaction with promoters through chromatin looping. Created in BioRender (https://BioRender.com/s38r6w8; accessed on 28 September 2025).

Enrichment for OA-associated SNVs in genomic regions functionally annotated as enhancers, categorizes OA as “enhanceropathies”, a group of diseases where disruption of enhancer–promoter interactions results in aberrant expression of a network of effector genes that drive disease pathogenesis [25]. Active enhancers are genomic regions with chromatin highly accessible to TFs. Typically, enhancers amplify the recruitment of the transcriptional machinery and increase gene expression by physical interaction with promoters through chromatin looping (Figure 4). The identification and functional characterization of enhancers is based on specific histone PTMs, chromatin accessibility, and 3D chromatin conformation. Thus, the genome-wide mapping of the OA-specific histone PTMs, chromatin accessibility, and 3D chromatin interactions between enhancers and promoters in OA-relevant cells is critical for the discovery of pathogenetic gene networks [24,45].

Histones contain flexible and charged N-terminal tails which are amenable to PTMs (e.g., acetylation and methylation), that determine the chromatin configuration and restrict or allow TF binding within enhancers and promoters [61]. Among the histone PTMs, the most extensively studied are acetylation and methylation of histone 3 (H3) at various lysine (K) residues [19,23,61]. Lysine acetylation neutralizes the charges on histone tails, weakens their binding to DNA, and increases the accessibility of binding motifs to TF, creating a permissive microenvironment for active transcription [23]. The impact of histone methylation on gene expression (activation or silencing) depends on the location (position of methylated K residue) and the number of methyl groups added [61]. For example, monomethylation of K4 on H3 (H3K4me1) at enhancer regions indicates enhancers in a poised state, suggesting a profound effect on gene expression [62]. Acetylation of K27 on H3 (H3K27ac), H3K4me1, an “open chromatin state”, and attachment of CCCTC-binding factor (CTCF) represent classic chromatin signatures indicating active enhancers with potential for enhancer–promoter looping [63,64]. The National Institute of Health (NIH) Roadmap Epigenomics Program has created the most comprehensive map of the histone PTM landscape, with topological and functional annotation of enhancers across >100 primary human cell types, including cell types from articular joints [65]. Articular cartilage is a tissue with low cellular abundance [66], restricting the feasibility of ChIP-based assays for high-quality genome-wide analysis of histone PTMs in primary chondrocytes derived from OA patients. Thus, it is not surprising that histone PTMs in human OA have been studied primarily with targeted approaches for specific genes, and genome-wide dynamic changes during disease have not yet been systematically assessed [67].

In contrast, the low cell numbers required for ATAC-seq have enabled the genome-wide characterization of chromatin accessibility in OA-relevant tissues [33,68,69,70,71]. Comparisons between damaged and intact cartilage from patients with end-stage OA havve identified genomic regions annotated as enhancers with differential chromatin accessibility [68]. These cartilage damage-specific enhancers were enriched with OA-associated SNVs and were correlated with differential expression of genes involved in chondrocyte differentiation and endochondral ossification. Inflammatory mediators, such as interleukin-1 (IL-1) and tumor necrosis factor (TNF), primarily derived from synovium and infrapatellar fat pad, are involved in OA pathogenesis [72]. In this context, another ATAC-seq study used a chondrosarcoma cell line as an in vitro tool to test the hypothesis that chromatin accessibility at OA-associated genomic regions is dynamically regulated by inflammatory signals [73]. With the caveat that a chondrosarcoma cell line is a transformed chondrocyte and thus may not accurately reflect the behavior of chondrocytes in the setting of OA, the study found that IL-1 impacts the expression of OA-relevant genes such as *matrix metallopeptidase 13* (*MMP13*) by disrupting chromatin accessibility and TF binding at genomic regions operating as enhancers for effector genes.

Mapping of the 3D chromatin organization enables the identification of enhancer–promoter interaction loops and facilitates the prioritization of effector genes that drive pathogenic transcriptional networks in OA-relevant tissues [25]. The first comprehensive maps of chromatin conformation in chondrocytes from OA patients were constructed very recently with the application of chromatin conformation capture technologies [33,74,75]. Integrated analysis of Hi-C and GWAS data revealed 10 OA-associated SNVs localized within chromatin loops of active enhancers interacting with promoters and identified two novel high-confidence effector genes, *pregnancy associated plasma protein A* (*PAPPA*) and *sprout RTK signaling agonist 4* (*SPRY4*) [75]. Notably, PAPPA is a metalloproteinase that increases the bioactivity of insulin-like growth factor-1 (IGF-1), which is involved in the repair of damaged chondrocytes [76]. Another study using ATAC-seq and Hi-C in primary human articular chondrocytes cultured with fibronectin fragment (FN-f), a validated ex vivo model of the OA chondrocyte phenotype, discovered gained enhancer–promoter loops and increased expression of previously discovered effector genes [33]. Overall, these studies on chromatin accessibility and 3D conformation support a model where OA is driven by aberrant gene expression networks due to disruptive effects of the OA-associated SNVs on long-range physical interactions between enhancers and promoters of effector genes [25].

## 6. The Chromatin Landscape and the “Developmental Origin” of OA

Emerging data on the temporal changes of epigenetic marks and chromatin states during skeletogenesis support the concept that developmental factors early in life may affect the risk of OA onset and progression later in life [71]. It is well known that events occurring during development determine the shape of the joint and the composition of articular cartilage and thus influence the risk of OA in adult life by impacting the biomechanical properties (e.g., the weight-bearing capacity and the resilience of the joints to withstand stress) throughout life [77]. Development of human limbs and synovial joints is a dynamic process that starts between four and eight post-conception weeks (pcw) [78]. This is a fine-tuned process that requires a spatiotemporally regulated expression of gene transcripts essential for skeletogenesis. According to the concept of a developmental origin for OA, the functional cooperation between chromatin dynamics and TFs regulates the unfolding of the development-specific gene expression program, defines the outcome of skeletogenesis, and ultimately determines the risk for OA.

**Figure 5:**
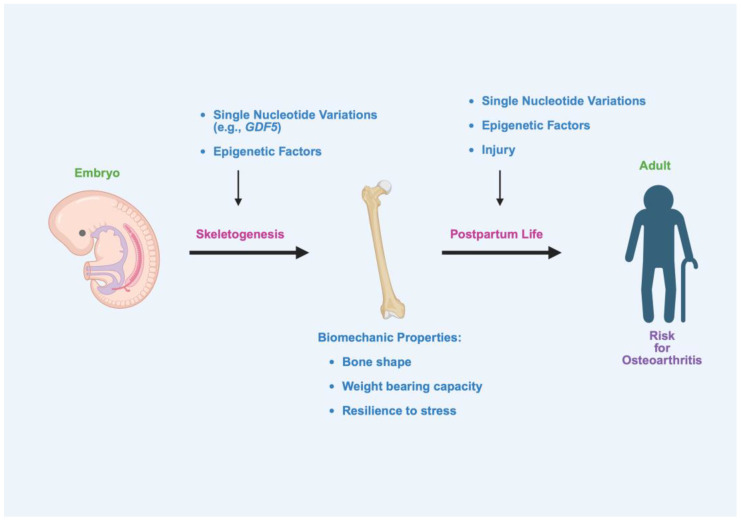
According to the “developmental origin hypothesis”, the risk for developing osteoarthritis (OA) in adult life is impacted by genetic (single nucleotide variations; SNVs) and epigenetic factors that operate during embryonic development and influence skeletogenesis. These factors determine the biomechanical properties of individual bones by influencing the shape, weight-bearing capacity, and resilience of bones to stress. SNVs and epigenetic dysregulation of *growth differentiation factor five* (*GDF5*) during skeletogenesis may increase the risk for developing OA by a potential impact on the bone shape. The ultimate risk of developing OA is also determined by SNVs and epigenetic factors operating during the postpartum life, as well as by injuries accumulated later in life. Created in BioRender (https://BioRender.com/g01oivm; accessed on 28 September 2025).

The overwhelming majority of studies aimed at delineating the genetic and molecular pathways involved in OA pathogenesis have focused on the analysis of adult tissues. The first study aiming to systematically pressure-test the “developmental origin hypothesis” for OA, compared the DNA methylation status and chromatin accessibility between fetal cartilage, fetal limb tissues, and adult cartilage [79]. When comparing fetal to adult cells, 85% of the tested mQTLs that were discovered in adult cells were replicated in the fetal cells. This finding might be just a coincidental correlation with no developmental consequences but may also suggest that OA-associated epigenetic mechanisms can potentially operate from the earliest stages of life. A subsequent study in developing human chondrocytes identified 24 loci at which OA-associated SNVs colocalize with mQTLs, indicating the presence of developmental epigenomic changes that potentially contribute to the risk of OA later in adult life [59]. Along the same lines, a recently published study harnessed ATAC-seq and gene expression data for 30 different cell types from the human skeletal development atlas [22]. In support of the “developmental origin hypothesis” (Figure 5), chondrocyte, osteoblast, and tenocyte development were associated with emergence of OA later in adult life. *GDF5* is a gene that plays quintessential roles in knee development across mammals by regulating the knee shape and is at the same time a well-established genetic locus associated with OA [80]. This is an example of a genetic factor that impacts the geometry of the knee during development by disrupting gene expression and may lead to OA during adult life due to abnormal joint biomechanics.

## 7. Chromatin as a Guide for Drug Discovery in OA: Opportunities and Challenges

Detailed mapping and functional characterization of the OA-associated chromatin conformation and epigenetic disruptions may accelerate the discovery of disease-modifying drugs for OA in multiple ways [24]. First, understanding the OA-specific chromatin landscape may facilitate the identification and prioritization of high-confidence effector genes. Through the integration of datasets from OA-associated chromatin maps, the pool of putative eGenes has recently expanded to 700, and emerging evidence implicates in OA pathogenesis a constellation of potentially druggable cellular and molecular pathways, even beyond cartilage homeostasis [22]. Notably, up to 10% of the OA-associated genes express proteins that are targeted by already approved drugs. These data offer opportunities for drug repurposing and pave the way for preclinical or early clinical development efforts testing, in the context of OA, drugs approved for other indications. In addition, since OA is a polygenic disease with pathogenetic and clinical heterogeneity, refining the disease endotype-specific chromatin and epigenetic signatures may guide precise and individualized therapeutic approaches.

Secondly, epigenetic modifications are reversible and there is proof of mechanism, primarily from oncology, regarding the druggability of the molecular apparatus involved in the addition (“writers”), removal (“erasers”), and reading (“readers”) of DNA methylation and histone PTMs [81]. Targeted deletion in murine articular chondrocytes of *Dnmt3b*, a gene encoding the enzyme that maintains DNA methylation and preserves homeostasis in articular cartilage, resulted in early onset OA [82]. The protective effects of DNMT3B are mediated through its impact on genes involved in chondrocyte metabolism and cartilage ECM turnover. In contrast, knock out of *tet1*, a gene encoding a DNA demethylation enzyme, protected from OA progression, and inhibition of TET1 enhanced the chondrogenic potential of adult skeletal stem cells (SSCs) [83,84]. In this context, pharmacologic or genetic manipulation of the DNMT3B/TET-1 balance appears as an attractive disease-modifying approach for OA.

Pharmacologic targeting of histone methylation and acetylation has recently emerged as a promising strategy to restore the aberrant gene expression networks that may drive OA pathogenesis [34,61]. A series of studies in articular chondrocytes have identified the regulation of H3K79 methylation as a critical epigenetic player in OA pathogenesis. Genetic deletion of *disruptor of telomeric silencing 1-like* (*DOT1L*), a gene encoding the major H3K79 methyltransferase, is associated with increased progression of age-related and post-traumatic OA [85,86,87]. In contrast, restoration of H3K79 methylation by pharmacologic inhibition of histone lysine demethylase 7A/B (KDM7A/B) protected against OA [88]. Thus, fine-tuning the opposing functions of DOT1L and KDM7A/B either by activating DOT1L or inhibiting KDM7A/B could be a promising disease-modifying therapeutic strategy for OA. On the other hand, preliminary studies in articular chondrocytes suggest that pharmacologic inhibition of histone deacetylases (HDACs) exhibit chondroprotective potential [89,90].

Pharmacologic manipulation of DNA methylation and histone PTMs impacts a wide range of gene programs in multiple cell types, including cells not relevant for disease pathogenesis. In addition, histone modifiers may have effects beyond histone targets [24,34,61]. Thus, potential safety concerns due to lack of specificity or off-target effects, is a major challenge for all types of treatments that target epigenetic modifications [91]. Clustered regulatory interspaced short palindromic repeats (CRISPR)-based technologies have been developed recently, aiming to overcome the issue of specificity and mitigate the concerns on safety. Molecular engineering has allowed the generation of catalytic deactivated CRISPR-associated (dCas) proteins, which maintain DNA-binding capacity and are fused with epigenetic modifiers, such as DOT1L, TET-1, or HDACs [92]. These dCas-based technologies pave the way for precise epigenetic editing at the desired genomic regions and may allow targeted transcriptional regulation of disease-relevant genes in disease-relevant cells.

## Data Availability

No new data were created or analyzed in this study.
